# Treating vulvovaginal atrophy/genitourinary syndrome of menopause: how important is vaginal lubricant and moisturizer composition?

**DOI:** 10.3109/13697137.2015.1124259

**Published:** 2015-12-26

**Authors:** D. Edwards, N. Panay

**Affiliations:** ^a^ Claridges Barn, Charlbury Road, Chipping Norton, Oxon, UK; ^b^ Queen Charlotte’s & Chelsea Hospital and Chelsea & Westminster Hospital, and Honorary Senior Lecturer, Imperial College, London, UK

**Keywords:** Cytotoxicity, genitourinary syndrome of menopause, lubricant, moisturizer, osmolality, vaginal dryness, vulvovaginal atrophy

## Abstract

Vaginal dryness is a common condition that is particularly prevalent during and after the menopause, and is one of the symptoms of vulvovaginal atrophy/genitourinary syndrome of menopause. The impact of vaginal dryness on interpersonal relationships, quality of life, daily activities, and sexual function can be significant, but is frequently underestimated. Furthermore, barriers exist to treatment-seeking, and this condition is often underreported and undertreated. Greater education about vaginal dryness and the range of available treatments is essential to encourage more women to seek help for this condition.

Personal lubricants and moisturizers are effective at relieving discomfort and pain during sexual intercourse for women with mild to moderate vaginal dryness, particularly those who have a genuine contraindication to estrogen, or who choose not to use estrogen. However, there is a distinction between lubricants and moisturizers, and notable differences between commercially available products. Women should be advised to choose a product that is optimally balanced in terms of both osmolality and pH, and is physiologically most similar to natural vaginal secretions. A series of recommendations for the use of vaginal lubricants and moisturizers, either on their own or in combination with systemic or topical hormone replacement therapy, is presented.

## Introduction

Vaginal dryness is prevalent among women of all ages, but is particularly common during and after the menopause^1–4^. Dryness is usually one of many symptoms reported by women as a result of vaginal or vulvovaginal atrophy (VVA). The term ‘genitourinary syndrome of menopause (GSM)’ has recently been proposed in reference to genitourinary tract symptoms related to the menopause, which is aimed at making the description of symptoms more inclusive and more user-friendly^5^^,^ 6. Reported prevalence rates of vaginal dryness due to VVA or GSM vary, but it is estimated that approximately 15% of premenopausal and up to 57% of postmenopausal women experience this condition^3^.

Although culturally accepted norms and practices regarding lubrication during sex differ between countries, women are typically expected to achieve a moderate amount of vaginal lubrication during sex^7^. Women themselves report that they prefer vaginal–penile intercourse to feel wetter, feel that they are more easily orgasmic when sex is wetter, and believe their partner prefers sex to feel more wet than dry^8^. Therefore, it is perhaps unsurprising that a lack of natural lubrication is one of the more commonly encountered sexual problems in the clinical care setting^9^.

The causes of decreased vaginal lubrication are numerous and include advancing age, hormonal changes, menopause, breastfeeding, stress, conditions such as diabetes, inflammatory bowel disease, chronic heart failure and multiple sclerosis^10^, and iatrogenic causes such as radiation and chemotherapy treatment^9^ and antidepressant use^11^.

There is an association between vaginal dryness and painful intercourse^12^, which is estimated to affect around half of all women at some point in their lives^13^, and inadequate lubrication is a common cause of dyspareunia (i.e. recurrent or persistent pain with sexual activity that causes marked distress)^14^.

The female sexual response cycle is initiated by neurotransmitter-mediated vascular and non-vascular smooth muscle relaxation, resulting in increased pelvic blood flow, vaginal lubrication, and clitoral and labial engorgement. These mechanisms are mediated by a combination of neuromuscular and vasocongestive events. Physiological impairments that interfere with the normal female sexual response can cause diminished sexual arousal, libido, vaginal lubrication, genital sensation, and the ability to achieve orgasm^15^. During the reproductive years, estrogen plays a key role in maintaining the normal vaginal environment. As estrogen levels fall during menopause, vaginal atrophy and thinning and inflammation of the vaginal walls and vulval tissues occur, which can result in decreased vaginal lubrication.

VVA is a widespread condition, with symptoms affecting around half of all peri- and postmenopausal women^16^. These symptoms can have a substantial negative impact on interpersonal relationships, quality of life, daily activities, and sexual function^17^. The REal Women's VIews of Treatment Options for Menopausal Vaginal ChangEs (REVIVE) survey in 3046 postmenopausal women with VVA symptoms in the US found the most common symptoms to be dryness (55% of participants), dyspareunia (44%), and irritation (37%), and these symptoms affected enjoyment of sex in over half (59%) of participants^17^. Symptomatic vaginal atrophy can also occur in younger women due to hypothalamic amenorrhea, hyperprolactinemia, lactation, and use of antiestrogen medications^18^. Atrophic symptoms affecting the vagina and lower urinary tract are often progressive and frequently require treatment^19^.

The symptoms of VVA may be successfully managed by a variety of prescription and over-the-counter (OTC) treatments, with choice of therapy dependent on symptom severity, the effectiveness and safety of the therapy for the individual patient, and patient preference. Available treatments include personal lubricants and moisturizers, topical vaginal estrogen, hormone therapy, and the selective estrogen receptor modulator, ospemifene (indicated for dyspareunia)^20^. Some women are reluctant to use vaginal estrogen, due to safety concerns^21^. For these women, personal moisturizers and lubricants are often recommended. Lubricants may relieve vaginal dryness and discomfort during sexual activity, providing short-term relief from vaginal dryness and dyspareunia^22^^,^ 23. Vaginal moisturizers are intended to be used primarily for the relief of vaginal dryness on a day-to-day basis, to provide comfort and offer long-term benefits. Vaginal moisturizers are classified as Class IIa Medical Devices by the Medicines and Healthcare products Regulatory Agency, based on the intended duration of their use (vaginal moisturizers are intended to be present in the body for longer than 60 min, but a single application should not last longer than 30 days). Lubricants may or may not be classified as medical devices, depending on their individual claims.

In women with breast cancer, the main side-effects of treatment, such as vasomotor symptoms and impaired sexual functioning, are related to premature menopause due to chemotherapy and/or anti-hormonal therapy^24^. For example, treatment with aromatase inhibitors is associated with vaginal dryness and atrophy, which are frequently accompanied by painful intercourse and decreased libido^25^. The use of hormones (e.g. local or systemic estrogen) poses a potential risk in patients with estrogen-dependent breast cancer, including those receiving antiestrogen adjuvant therapies. Therefore, the recommended first-line therapy for vaginal dryness and dyspareunia is usually non-hormonal treatments, such as personal moisturizers and lubricants^25^^,^ 26.

Many women perceive vaginal dryness and discomfort as having a substantial negative impact on their lives, particularly with regard to sexual intimacy, their ability to have a loving relationship, and overall quality of life^20^^,^ 27, although the degree to which women are bothered by these conditions varies considerably across countries^2^.

Despite the availability of various treatment options, underreporting and undertreatment of vaginal dryness are common, and only a minority of women seek medical help^19^. In a survey conducted in the UK among a representative sample of women aged from 55 to over 85 years (*n* = 2045), 33% of the study sample who reported dyspareunia and/or vaginal dryness did not seek professional advice, and 36% resorted to an OTC remedy^28^. Similar findings were also reported from the Vaginal Health: Insights, Views and Attitudes questionnaire-based survey, in which 37% of women with symptoms relating to vaginal discomfort did not consult a health-care professional (HCP), and 40% waited 1 year or more before doing so^27^.

Evidence suggests that a lack of awareness among women about the physiological changes associated with the menopause and the availability of effective and well-tolerated treatments, reluctance to discuss symptoms with HCPs, safety concerns, inconvenience, and inadequate symptom relief from available treatments are potential barriers to seeking and using treatment^16^^,^ 17.

Although personal lubricants and moisturizers have demonstrated effectiveness, they differ in terms of their composition, and certain individual components may be of concern in specific situations. Therefore, it is important to choose the most appropriate lubricant or moisturizer to best suit the needs of the individual patient.

This review provides an overview of commonly available personal lubricants and moisturizers, discusses their composition and advantages and disadvantages, and explores associations between ingredients and potential biological effects. It will also highlight the unmet needs of women with vaginal dryness, and how these can be addressed.

## Personal lubricants and moisturizers

Personal lubricants and moisturizers are effective in relieving discomfort and pain during intercourse for women with mild to moderate vaginal dryness, particularly women who are not suitable for vaginal estrogen therapy, or who do not wish to use it. Both lubricants and moisturizers reduce the friction associated with thin, dry genital tissue that can occur as a result of VVA/GSM. The main difference between vaginal moisturizers and lubricants is in their intended use.

### Personal lubricants

A wide variety of personal lubricants are commercially available, either as water-, silicone-, mineral oil-, or plant oil-based products, and are applied to the vagina and vulva (and the partner’s penis if required) prior to sex. Lubricants act rapidly to provide short-term relief from vaginal dryness and related pain during sex. They are particularly beneficial for women whose vaginal dryness is a concern only or mainly during sex.

Water-based lubricants have the advantage of being non-staining. An internet-based, double-blind, prospective daily diary study also found that water-based lubricants are associated with fewer genital symptoms in women, compared with silicone-based lubricants^29^. Excipients acting as humectants, emollients and preservatives are added to water-based lubricants to achieve viscosity, alter water activity, and prevent bacterial contamination, and these will have an impact on pH and osmolality values, which are discussed in further detail in a later section. Oil- and silicone-based lubricants are not addressed in this article because they do not have a pH or an osmolality value, as they contain no water. Some prescription and OTC water-based lubricants will also contain some, or all, of the following: glycerine, propylene glycol, parfum, sweeteners, warming agents and parabens. It is worth pointing out that a few well-known vaginal lubricants do not list the ingredients on the pack.

The World Health Organization (WHO) recommends that additional lubricants are used with condoms for women in the menopause and postmenopause as well as in other groups, such as female sex workers and men who have sex with men^30^.

### Vaginal moisturizers

Vaginal moisturizers rehydrate dry mucosal tissue and are absorbed into the skin and adhere to the vaginal lining, thereby mimicking natural vaginal secretions. Vaginal moisturizers are intended to be used for the non-hormonal alleviation of vaginal dryness/atrophic vaginitis/vaginal atrophy and they are applied regularly, from every day to once every 2–3 days. Their frequency of use is directly proportional to the severity of atrophy (i.e. the more severe the atrophy, the more frequent the application), and their effects are more long-term than those of lubricants, lasting 2–3 days. Vaginal moisturizers provide this longer relief by changing the fluid content of the endothelium and lowering vaginal pH^31^, thereby maintaining vaginal moisture and acidity. They are therefore particularly beneficial not only for women with symptoms of VVA/GSM that cause pain during sexual activity, but also for women who are not necessarily sexually active, but experience day-to-day discomfort. As vaginal moisturizers are intended to moisturize the mucosa, the majority contain water. In order for the water to adhere to the mucosa, they also contain either plant-based or synthetic polymers. In addition, they contain a wide range of other excipients to provide the appropriate viscosity, pH buffering and preservation, and it is these additional ingredients (and some synthetic polymers) that will affect the pH and osmolality of the moisturizer.

A list of commercially available vaginal moisturizers and lubricants, with their ingredients, pH and osmolality values, is provided in Table 1.

**Table 1.** Commonly used personal lubricants available world-wide. Certified organic ingredients are in bold.

*Name*	*Ingredients*	*pH*	*Osmolality (mOsm/kg)*
*Moisturizers*			
Canesintima Intimate Moisturiser	aqua, glycerin, glyceryl polymethacrylate, capryloyl glycine, sorbitol, acrylates/C10-30 alkyl acrylate crosspolymer, sodium hyaluronate, sodium benzoate, sodium hydroxide, galactoarabinan, butylene glycol/*Camellia japonica* leaf/flower extract, tetrasodium EDTA, p-ansic acid, levulinic acid	5.63^a^	846^b^
Gynomunal Vaginal Moisturising Gel	hop extract (*Humulus lupulus*), tocopherol acetate (vitamin E), purified water, propylene glycol, denatured ethanol, soya lecithin (E322), carbomer, methyl-4-hydroxybenzoate (E219), cholesterol, imidazolidinylurea, triethanolamine, sodium edetate, hyaluron	5.84^a^	>2000^b^
Hyalofemme Vaginal Hydrating Gel	hydeal-D (hyaluronic acid derivative), propylene glycol, carbomer, methyl p-hydroxybenzoate, propyl p-hydroxybenzoate, sodium hydroxide, purified water	4.88^a^	1729^b^
Regelle Long-Lasting Vaginal Moisturiser	purified water, polycarbophil, glycerol, mineral oil, hydrogenated palm oil glycerides, carbopol 974P, sorbic acid	2.88^a^	2012^b^
Replens MD Longer-Lasting Vaginal Moisturiser	purified water Ph. Eur. 78.64% w/w, glycerin, mineral oil, polycarbophil, carbomer homopolymer type B, hydrogenated palm oil glyceride, methylparaben, sorbic acid, sodium hydroxide	2.95^a^	2011^b^
Sylk Natural Intimate Moisturiser	water, extracts of kiwifruit plant and citrus seed, xanthan gum, vegetable glycerin, citric acid, potassium sorbate, sodium citrate	4.47	877^b^
Yes Vaginal Moisturiser^f^	aqua, ***Aloe barbadensis*leaf juice**, **guar gum**, **locust bean gum**, **flax seed extract**, xanthan gum, sodium chloride, citric acid, potassium sorbate, phenoxyethanol	4.15	250
*Lubricants*			
Astroglide Gel Lubricant	purified water, glycerin, hydroxyethylcellulose, chlorhexidine gluconate, methylparaben, glucono delta lactone, sodium hydroxide	4.38	6100^b^^,^^c^
Astroglide Ultra Gentle Sensitive Skin Lubricant	purified water, xylitol, hydroxyethylcellulose, *Aloe barbadensis* leaf juice, pectin, *Chamomilla recutita* (Matricaria) flower extract, phenoxyethanol	4.56^a^	945^b^
Balance Activ Menopause Vaginal Moisturising Lubricant	phosphate-buffered saline, sodium hyaluronate (hyaluronic acid), phenoxyethanol, methylparaben	5.64^a^	309
Bioglide Natural Lubricant	glycerin, aqua, sodium lactate, xanthan gum, levulinic acid, sodium levulinate	4.99^a^	>2000^b^
Durex Play Feel Lubricant	purified water, propylene glycol, hydroxyethylcellulose, benzoic acid, sodium hydroxide	5.48^a^	1563^b^
Durex Sensilube Hydrating Intimate Gel	aqua, phenoxyethanol, polyacrylamide, hydroxyethylcellulose, methylparaben, ethylparaben, propylparaben, citric acid	5.99^a^	16^b^
Good Clean Love Lubricant	***Aloe barbadensis*leaf juice**, xanthan gum, agar, lactic acid, potassium sorbate, sodium benzoate, natural flavor	4.73^a^	240
Higher Nature V Gel Aloe Vera Lubricant	*Aloe barbadensis (Aloe vera)*, purified aqua, glycerine, xanthan gum, panthenol, allantoin, sodium benzoate, potassium sorbate, *Calendula* (marigold), retinyl palmitate (vitamin A), tocopheryl acetate (vitamin E), zinc gluconate, citric acid	4.09	1646^b^
ID Glide Lubricant	water, glycerin, propylene glycol, cellulose polymer, polyethylene oxide, sodium benzoate, methylparaben, carbomer 981, tetrahydroxypropyl ethylenediamine, diazolidinyl urea, EDTA	5.20^a^	3200^b^^,^^c^
Intimate Organics Lubricant	water, propanediol, cellulose gum, sodium benzoate, citric acid, alcohol denat, *Lycium barbarum* fruit extract, *Cymbopogon schoenanthus* extract, *Aloe barbadensis* leaf extract	4.86^a^	>2000^b^
Intimy Lubricant	hydroxyethylcellulose, glycerin, cocoalkonium chloride, citric acid	6.19^a^	1501^b^
Klick Natural Glide Lubricant	aqua (water), propylene glycol, glycerin, hydroxyethylcellulose, phenoxyethanol, lactic acid, sodium saccharin, benzoic acid, sodium hydroxide, dehydroacetic acid	4.84^a^	>2000^b^
KY Jelly Lubricant	water, glycerin, hydroxyethylcellulose, chlorhexidine gluconate, gluconolactone, methylparaben, sodium hydroxide	4.49	2007^b^
Phyto Soya Vaginal Gel	aqua, glycerin, paraffinum liquidum, sodium hydroxide, carbomer, soya extract (glycine max), phenoxyethanol, methylparaben, propylparaben, isobutylparaben	4.94^a^	1557^b^
Pjur Med Natural Glide Personal Lubricant	aqua (water), glycerin, xanthan gum, benzyl alcohol, sodium benzoate, potassium sorbate, citric acid	4.41	>2000^b^
Pjur Woman Nude Lubricant	aqua (water), propylene glycol, ethoxydiglycol, hydroxypropyl guar hydroxypropyltrimonium chloride, hydroxyethylcellulose, sodium saccharin, citric acid	4.42	>2000^b^
RepHresh Vaginal Gel	purified water, glycerin, polycarbophil, carbomer homopolymer type B, ethylparaben sodium, methylparaben sodium, propylparaben sodium, sodium hydroxide	3.46^a^	1914^b^
Ritex Sensitiv Gel	aqua, glycerin, propylene glycol, hydroxyethylcellulose, sodium lactate, lactic acid	4.04	>2000^b^
Sass Intimate Dryness Gel	aqua, glycerin, butylene glycol, ammonium acryloydimethyl taurate, VP copolymer, panthenol, xylitylglucoside, anhydroxylitol, PEG-40 hydrogenated castor oil, allantoin, sodium benzoate, xylitol, *Aloe barbadensis* leaf juice, potassium sorbate, parfum, disodium EDTA	4.99^a^	>2000^b^
Simply Slick Personal Lubricating Lotion	castor oil, purified water, jojoba oil, vegetable glycerin, pectin, *Stevia*, optiflo H370VF	6.68^a^	>2000^b^
System Jo Personal Lubricant	water (aqua), **locust bean gum**, ***Aloe barbadensis*leaf juice**, ***Euterpe oleracea*(Acai) pulp powder**, xanthan gum, **citrus extract**	5.86^a^	61
Yes But Anal Lubricant	aqua, ***Aloe barbadensis*leaf juice**, **guar gum**, **locust bean gum**, xanthan gum, honeysuckle flower extract, sodium chloride, citric acid, sodium hydroxide	7.78^a^^,^^d^	330^d^
Yes Baby Sperm-Friendly Lubricant	aqua, ***Aloe barbadensis*leaf juice**, **guar gum**, **locust bean gum**, xanthan gum, honeysuckle flower extract, sodium chloride, citric acid, sodium hydroxide	7.65^a^^,^^e^	333^e^
Yes Baby Vaginal-Friendly Lubricant	aqua, ***Aloe barbadensis*leaf juice**, **guar gum**, **locust bean gum**, **flax seed extract**, xanthan gum, sodium chloride, citric acid, potassium sorbate, phenoxyethanol	4.22	249
Yes Water-Based Intimate Lubricant	aqua, ***Aloe barbadensis*leaf juice**, **flax seed extract**, **guar gum**, **locust bean gum**, xanthan gum, sodium chloride, potassium sorbate, citric acid, phenoxyethanol	4.08	154

^a^, Values are outside the normal vaginal pH range of 3.8–4.5^30^;

^b^,  values represent hypo-osmolar (< 32 mOsm/kg) or hyperosmolar preparations that exceed the ideal osmolality threshold of 380 mOsm/kg recommended by the World Health Organization for a personal lubricant (most of which also exceed the real-world recommended threshold of 1200 mOsm/kg)^30^, and which therefore have the potential to cause irritation and/or damage to vaginal or rectal mucosa;

^c^,  osmolality value taken from Wolf 2012^68^;

^d^,  matches rectal pH (∼7.0) and osmolality;

^e^,  matches semen pH and osmolality;

^f^,  Class IIa in progress.

All ingredient lists were taken from products purchased June to August 2015. Product and batch numbers are available on request from The Yes Yes Company Ltd. Osmolality and pH testing methods are described in the online Supplemental Material S1 (http://dx.doi.org/10.3109/13697137.2015.1124259).

## Perceptions and attitudes towards lubrication and lubricants

The use of commercially available personal lubricants is common among adult women^32^ and their perceptions of lubricants and lubricant use are generally positive, although women in their forties report more positive perceptions of lubricants than women under the age of 30^8^.

Younger women and those without sexual dysfunctions, such as vaginal dryness or dyspareunia, are more likely to use lubricants to enhance the sexual experience and make sex more comfortable, fun, and pleasurable, while older women and those with dyspareunia or symptoms of VVA/GSM report using lubricants to reduce or alleviate discomfort and pain^12^^,^ 32.

Evidence suggests that the use of personal lubricants is more frequent as part of partnered intercourse and sexual play or foreplay, compared with other solo and partnered sexual behaviors^32^. A study has shown that the use of water- or silicone-based lubricants is associated with higher ratings of sexual pleasure and satisfaction for penile–vaginal sex, as well as solo sex, compared with no lubricant use^29^.

## Characteristics to consider when choosing a personal lubricant

Personal lubricants can be composed of a variety of ingredients as shown in Table 1. The pH, osmolality, and presence of certain individual components in a personal lubricant may be associated with detrimental biological effects, meaning that some products are less suitable for use in specific situations and populations, such as couples trying to conceive or those at higher risk of exposure to sexually transmitted infections.

### Links between osmolality, pH and cytotoxicity of lubricants

In the vagina, a multispecies microbiota usually associated with bacterial vaginosis occurs as a dense biofilm, while a lactobacilli-dominant microbiota is sparsely distributed on the vaginal epithelium^33^. Estrogen stimulates the proliferation of lactobacilli, reduces pH, and prevents vaginal colonization of Enterobacteriaceae, which are the main pathogens of the urinary tract. In postmenopausal women, a reduction in estrogen leads to declining lactobacilli numbers and a subsequent rise in vaginal pH, which provides favorable conditions for colonization of the vagina with Enterobacteriaceae from the rectum^34^.

**Figure 1.**
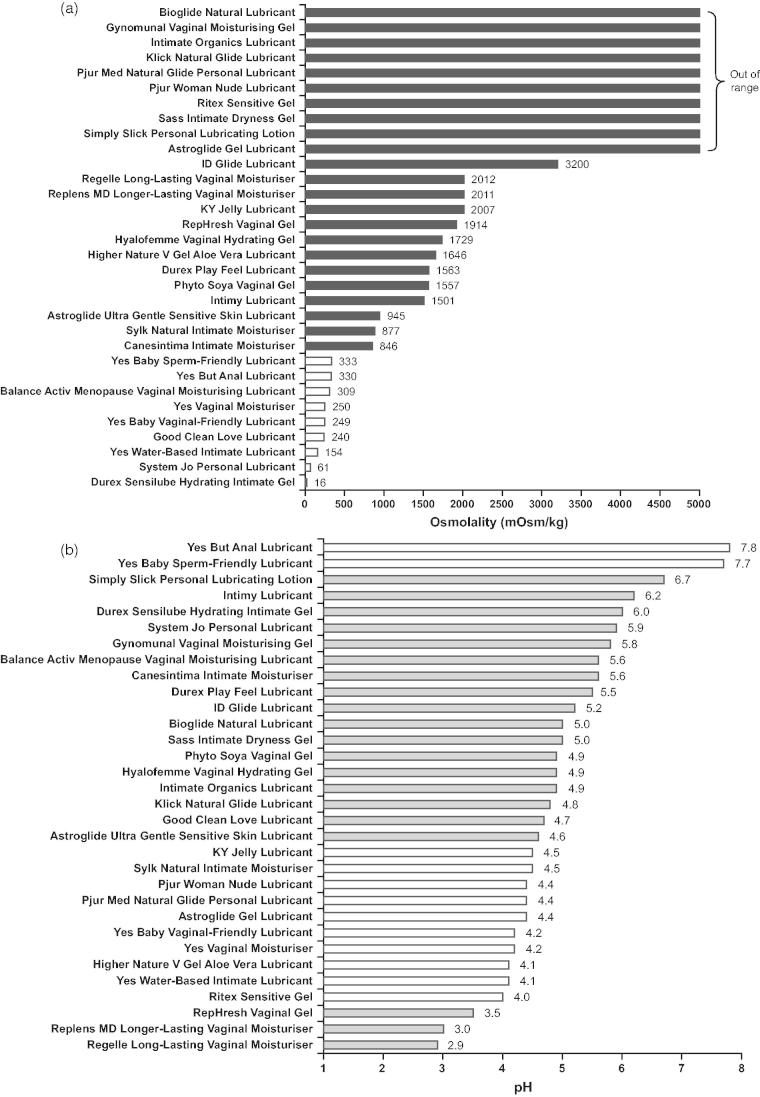
Osmolality (a) and pH (b) of a panel of personal lubricants and moisturizers available world-wide. Black bars represent hyperosmolar preparations that exceed the ideal osmolality threshold of 380 mOsm/kg recommended by the World Health Organization for a personal lubricant (most of which also exceed the real-world recommended threshold of 1200 mOsm/kg)^30^, and which therefore have the potential to cause irritation and/or damage to vaginal or rectal mucosa. In healthy adults, normal vaginal and rectal pH ranges are 3.8–4.5 and ∼7.0, respectively^30^. Gray bars represent preparations that are considered outside of these thresholds. Osmolality and pH testing methods are described in the online Supplemental Material S1 (http://dx.doi.org/10.3109/13697137.2015.1124259).

The osmolality of personal lubricants can vary widely, as shown in a recent evaluation of a panel of products available in Europe and North America (Figure 1a). Osmolality of personal lubricants has been the focus of recent advice from the WHO, in collaboration with the United Nations Population Fund and Family Health International^30^. The WHO recommends that the osmolality of a personal lubricant should not exceed 380 mOsm/kg, in order to minimize any risk of epithelial damage; however, because most of the commercially available preparations greatly exceed this value, an upper limit of 1200 mOsm/kg is generally deemed acceptable in practice^30^.

Greater osmolality of personal lubricants has been significantly correlated with increased potential to cause mucosal irritation and tissue damage in a slug mucosal irritation (SMI) assay. This assay is used as a sensitive measure of mucus membrane tolerance for vaginal microbicide products and carriers, and the degree of irritation in the assay can predict genital burning, heat and itching in humans^35^. *Arion lusitanicus* slugs were treated with lubricants over 5 days to quantify mucus production and tissue damage, allowing assignment of each product to an irritation potency category (i.e. none, mild, moderate or severe). Results showed hypo-osmotic lubricants (32–316 mOsm/kg) had no adverse effects, moderately hyperosmotic lubricants (Replens: 2143 mOsm/kg, KY Jelly: 2463 mOsm/kg) induced mild to moderate irritation, and a very hyperosmotic lubricant (Astroglide: 5848 mOsm/kg) caused severe irritation and tissue damage^35^.

High osmolality of personal lubricants has also been associated with cytotoxicity. In a prospective, comparative, *in vitro* study, incubating sperm with hyperosmolar lubricants (> 1000 mOsm/kg; Astroglide, KY Jelly, Replens) led to loss of motility and DNA integrity^36^. Exposure to hyperosmolar lubricants has also been shown *in vitro* to damage epithelial cell lines, and cervical and colorectal explant cultures^37^, and, when applied rectally in humans, hyperosmolar lubricants cause significant damage and denudation of the epithelium^38^.

Like osmolality, pH can vary widely among personal lubricant products (Figure 1b). In healthy adults, normal vaginal and rectal pH ranges are 3.8–4.5 and ∼7.0, respectively^30^, and the optimum requirements for both vaginal and rectal intercourse cannot be bridged in a single lubricant. Cunha and colleagues commented that 'outcomes of low pH are even less understood, but animal data suggest that values of 3 or less are unacceptable for human use'^39^. Therefore, clinicians need to be aware that some preparations do not meet this recommendation (see Table 1).

### Excipients in personal lubricants

Although cytotoxic effects associated with hyperosmolar lubricants have been demonstrated *in vitro* and in humans in several studies^36–38^, a recent study of 12 commercially available lubricants of varying pH and osmolalities failed to find a significant association between these criteria and cytotoxicity^39^. The authors suggested that individual components of the surveyed personal lubricants therefore may have a greater influence on cytotoxicity than pH or osmolality, and added that 'further specific toxicity testing using vaginal microbiota, namely *Lactobacillus* spp., is advisable'^39^.

#### Parabens

Parabens are included as preservatives in a variety of personal care, cosmetic and food products, and are found in some personal lubricants, such as KY Jelly, Replens and Astroglide. Parabens are weakly estrogenic compounds and there is some debate as to whether they present an endocrine-disrupting risk^40–42^. Parabens have been detected in breast tumors^43^, but direct associations with carcinogenesis or significant adverse effects in toxicology studies have not been convincingly demonstrated, and further research is needed.

#### Glycols

Glycol concentration is the primary factor determining osmolality for the majority of personal lubricants^30^. Glycols serve as humectants/emollients in lubricants, and glycerol/glycerine and propylene glycol are the most common. To maintain the osmolality of a personal lubricant at < 1200 mOsm/kg, the WHO advises that the concentration of glycerol should not exceed 9.9% mass fraction (w/w), and the concentration of propylene glycol (or a mixture of glycols) should not exceed approximately 8.3% mass fraction (w/w)^30^.

Apart from their key role in osmolality and mucosal irritation, glycols have also shown adverse effects in animal and *in vitro* studies. Vaginal application of glycerol monolaurate, glycerine, propylene glycol and PEG-8 all significantly increased susceptibility to herpes simplex virus 2 (HSV-2) in a mouse model^44^. An OTC personal lubricant containing propylene glycol, glycerine and methylparaben has also been shown to kill *Lactobacillus crispatus in vitro*, which is the dominant bacterial species in the vaginal microbiome that helps maintain a healthy mucosal barrier and acidic pH^45^. Indeed, recent personal lubricant use is associated with incident bacterial vaginosis outbreaks (adjusted odds ratio 11.75, 95% confidence interval 1.96–70.27), and this is thought to be related to the presence of glycerine and/or the microbicidal preservative chlorhexidine in the lubricant^46^. Low concentrations of glycerine/glycerol and their metabolites have also been shown to serve as a food source for *Candida albicans* 47.

#### Microbicides and preservatives

Microbicides in personal lubricants can cause epithelial damage and inflammation of the genital mucosa, alterations in the vaginal microbiome, and increased susceptibility to sexually transmitted infections. For example, early research showed that the microbicidal and spermicidal detergent, nonoxynol-9, caused rapid exfoliation of uterine and rectal epithelial cells in animal models^48^^,^ 49 and rectal epithelium in humans^50^. In animal studies, nonoxynol-9-associated irritation and mucosal damage was accompanied by increased susceptibility to infection by HSV-2^49^^,^ 51. In the SMI assay, nonoxynol-9-containing personal lubricants induced severe mucosal irritation, while products without nonoxynol-9 did not^52^. Nonoxynol-9-containing personal lubricants were also shown to remove rectal mucosa, which may promote rectal transmission of human immunodeficiency virus (HIV) and other sexually transmitted infections^53^. Consequently, the WHO does not recommend the use of nonoxynol-9-containing personal lubricants^30^. Today, nonoxynol-9 is used infrequently, mainly on condoms.

Certain preservatives are associated with negative effects on pathogen transmission. After a single vaginal administration, chlorhexidine caused cellular damage in a mouse chlamydia model, accompanied by a 100-fold increased susceptibility to infection versus without the detergent^54^. In a cell monolayer model, personal lubricants containing polyquaternium-15 significantly increased HIV-1 replication *in vitro* 55.

#### Effects on fecundity

The effect of personal lubricants and vaginal moisturizers on fecundity remains to be resolved^56^. *In vitro* studies have shown that some commonly used personal lubricants, such as Astroglide, KY Jelly and Replens, can greatly impair sperm motility, even at very low concentrations^36^^,^ 57–60. In one study, sperm exposed to KY Jelly became immobile after 60 min^59^, and a >10% increase in chromatin damage after lubricant exposure in sperm from healthy donors has also been reported^36^. In contrast, Pre-Seed was found to have no effect on sperm motility and did not disrupt sperm chromatin structure^36^. Similarly, DNA fragmentation was not observed in a more recent study of the effects of nine different products on sperm from men attending a fertility clinic^61^.

The negative consequences on sperm motility and viability may be attributed to the presence of constituents, such as glycerine, which can damage the flagellar membrane of sperm^62^ and profoundly inhibit sperm motility^59^, and the non-physiological pH and/or osmolality of certain lubricants. The optimum pH for sperm migration and survival in cervical mucus is 7.2–8.5^63^, and a reduction in sperm motility is observed at pH levels below 6^64^. Similarly, sperm motility is nearly abolished with increases in osmolality approaching 600 mOsm/kg^65^, and a physiologic osmolality of 270–360 mOsm/kg is best for sperm function^36^.

Despite many studies demonstrating harmful effects of personal lubricants on sperm *in vitro*, a large, prospective cohort study in couples trying to conceive (*n* = 296 women) found no evidence of reduced fecundity among lubricant users versus their counterparts who did not use lubricants (the lubricants used were not specified by the study participants)^56^. Further randomized, controlled studies are needed to determine the effects of personal lubricants on fertility.

## Key practical issues

The impact of vaginal dryness on quality of life is often underestimated. From the couple’s perspective, vaginal dryness can cause problems in relationships. It can accentuate erectile dysfunction in the male partner, and even cause single women to avoid entering into a relationship with a potential new partner.

Many women are reluctant to raise such problems with their HCP due to embarrassment and/or cultural reasons, and HCPs often do not proactively raise the issue in consultations because they are uncomfortable discussing sexual issues^6^. There is also a lack of knowledge about the range of effective treatment options that are available.

Two key challenges that need to be addressed are: (1) encouraging more women to report symptoms of VVA/GSM and (2) educating HCPs, i.e. nurses, general practitioners, sexual health specialists and gynecologists, to sensitively engage in conversation with their patients about these problems. Even though VVA/GSM is known to affect up to 50% of women postmenopausally^23^, evidence suggests that, if HCPs fail to ask the correct questions, only a small proportion of these women will proactively state that there is a problem.

Vulval irritation and contact dermatitis are common complaints among women^66^ and can be caused by exposure to hyperosmotic lubricants and excipients. However, identifying these as the causative agents and eliminating them can be challenging. There is also the potential for misdiagnosis, with vulval irritation commonly diagnosed as candidal vulvovaginitis (vaginal thrush)^67^. Misdiagnosis and prescribing or recommending products which then cause irritation may be associated with increased treatment costs, because patients may require additional medication.

The benefits of a good-quality lubricant/moisturizer are clear in women who have a genuine contraindication to estrogen, or in those who choose not to use estrogen; however, in our view, most women/couples can benefit from a good-quality lubricant, regardless of whether it is appropriate for the woman to use estrogen or not. There is likely to be a synergistic benefit to a good lubricant being used with estrogen or ospemifene, although this observation is based solely on clinical experience, and further research is needed to confirm it. The use of topical estrogen and lubricants at different times of the day is recommended, because it is possible that estrogen absorption may be impeded if applied immediately after using a lubricant. It is also recommended to delay intercourse until at least 1 hour after the application of estrogen, in order to avoid any possibility of transmission to the partner.

In our clinical practice, we encourage patients to choose a lubricant which is physiologically most similar to natural vaginal secretions (i.e. a 'body identical replacement'). Given the evidence presented in this paper and in our clinical experience, we believe it is vital that the chosen product is optimally balanced in terms of both osmolality and pH.

A public awareness campaign combined with education for HCPs would be valuable to highlight the differences between different lubricants and moisturizers and to encourage the routine use of a good-quality, 'body identical' lubricant by HCPs during examination and smear taking.

## Conclusion

Vaginal dryness is a common symptom in women with VVA/GSM and has a substantial negative impact on their sexual and overall quality of life. Despite this, barriers exist to treatment-seeking, and this condition is underreported and undertreated.

Women with VVA/GSM should be encouraged to discuss their symptoms, and HCPs should proactively raise this topic with their patients, in a sensitive manner. Greater education about vaginal dryness and the range of treatments available may urge women to seek treatment, with HCPs playing a pivotal role in this regard. Both primary and secondary HCPs need to proactively consider the possibility of VVA/GSM in patients who are not obviously seeking a gynecological opinion, such as patients with diabetes or those taking antidepressants. Clinical experience shows that, in general, patients are not insulted if sexual problems are discussed.

Personal lubricants and moisturizers are effective treatment options in the management of vaginal dryness with a variety of causes. However, differences exist between commercially available products. Given that non-physiological pH and osmolality, and the presence of excipients such as parabens and microbicides, are associated with a variety of proven or potential detrimental effects, the recommended safe values for pH and osmolality should be carefully ensured when choosing or prescribing a personal lubricant. This provides a stimulus for both regulatory authorities and manufacturers to work together in reformulating preparations to be more patient-friendly.

It is advised that women choose a product that is optimally balanced in terms of both osmolality and pH and is physiologically most similar to natural vaginal secretions. Table 2 contains a series of recommendations for the use of vaginal lubricants and moisturizers, either on their own or in combination with systemic or topical hormone replacement therapy.

**Table 2.** Recommendations for health-care professionals on vaginal lubricants and moisturizers for use on their own or in combination with hormone replacement therapy (systemic or topical).

*Symptom or situation*	*Recommendation*	*Rationale*
Urogenital atrophy, elevated vaginal pH, experiencing pain in daily life due to extreme dryness	Use a vaginal moisturizer with acidic pH and osmolality below the WHO ideal recommendation of 380 mOsm/kg	Rehydrate vaginal tissues and lower vaginal pH to minimize infection (e.g. bacterial vaginosis)
Dyspareunia (painful intercourse) caused by urogenital atrophy	Use a vaginal lubricant with acidic pH matched to vaginal pH and with osmolality below the WHO ideal recommendation of 380 mOsm/kg	Lubricate dry vaginal tissues without causing irritation and maintain or lower vaginal pH
Urogenital atrophy as a result of cancer treatment when HRT is contraindicated, or in combination with topical estrogen if still experiencing discomfort from atrophy	For daily comfort, use a paraben-free vaginal moisturizer with acidic pH and osmolality below the WHO ideal recommendation of 380 mOsm/kgFor sexual intercourse or for use with vaginal dilators, use a paraben-free vaginal lubricant with acidic pH matched to vaginal pH and osmolality below the WHO ideal recommendation of 380 mOsm/kg	Rehydrate vaginal tissues and lower vaginal pH to minimize infectionLubricate dry vaginal tissues without causing irritation and maintain or lower vaginal pHAvoid potential endocrine disruptors (i.e. paraben preservatives)
Trying to conceive and needing a lubricant	At ovulation, use a sperm-friendly lubricant	Lubricant is pH-matched and iso-osmotic to semen
Rectal/anal sex	Use a rectal lubricant that is condom-compatible with osmolality below the WHO ideal recommendation of 380 mOsm/kg and a pH matched to rectal pH	Reduce the risk of condom damage and resulting pathogen transmission, without irritating/damaging the rectal epithelium
Vaginal or rectal examination	Use a lubricant that is pH-matched for the vagina or rectum and has osmolality below the WHO ideal recommendation of 380 mOsm/kg	Reduce the risk of irritating/damaging the vaginal or rectal epithelium

HRT, hormone replacement therapy; WHO, World Health Organization.

## Supplementary Material

Supplementary_1124259.docx
