# Supplementary material for: Treating vulvovaginal atrophy/genitourinary syndrome of menopause: how important is vaginal lubricant and moisturizer composition?
Source: Climacteric. 2015 Dec 26;19(2):151–61. doi: 10.3109/13697137.2015.1124259 (PMC4819835; doi:10.3109/13697137.2015.1124259)
Supplement: Supplementary_1124259.docx [file icmt_a_1124259_sm8230.docx]

**Supplementary Material S1.** Osmolality and pH testing methodology.

### Osmolality measurements

The osmolalities of the test compounds were measured using a Model 3300 Advanced Micro-Osmometer (Advanced Instruments, Norwood, MA, USA) by the freezing-point method. The device was calibrated with a Clinitrol 290 reference solution (Advanced Instruments). Measurements were performed in duplicate (on 20 μL aliquots), and mean values were calculated.

### pH measurements

pH measurements were performed using a P601 pH meter (CONSORT nv, Turnhout, Belgium). The device was calibrated in a range between pH 5–9, with a control at pH 7 (measured pH 7.02). The pH of each sample was measured once.
